# Impact of a remdesivir formulary restriction by antimicrobial stewardship on drug utilization and cost at a safety-net community hospital during the COVID-19 pandemic

**DOI:** 10.1017/ash.2024.438

**Published:** 2024-10-07

**Authors:** Alfredo Jose Mena Lora, Dylan Huber, Ella Li, Romeen Lavani, Mirza Ali, Eden Takhsh, Rodrigo Burgos

**Affiliations:** 1 University of Illinois at Chicago, Chicago, IL, USA; 2 Saint Anthony Hospital, Chicago, IL, USA

## Abstract

Deploying novel COVID-19 therapies proved challenging amid rapidly evolving data, drug shortages, and conflicting guidelines. We established a remdesivir formulary restriction remdesivir to promote its evidence-based use. This intervention led to changes in remdesivir utilization patterns and cost savings. Formulary restrictions can play an important role in pandemic preparedness and response.

## Background

Remdesivir (RDV) was the first antiviral with clinical benefit against COVID-19 proven in a randomized placebo-controlled trial (RCT).^1,2^ RDV shortens hospitalization days for patients with COVID-19 requiring low-flow nasal cannula (NC). However, high-quality RCTs have shown no benefit for mechanically ventilated (MV) patients, and there is conflicting data on RDV effectiveness for patients requiring high-flow nasal cannula (HFNC) and non-invasive positive pressure ventilation (NIPPV).^1–5^ This led to differing recommendations from major guidelines and highly variable institutional use criteria across hospitals in the United States (US).^2–4^

Amid rapidly evolving data, drug shortages, and conflicting guidelines, deploying novel therapeutics during the COVID-19 pandemic proved challenging.^2–4^ Antimicrobial stewardship programs (ASP) can optimize antimicrobial use (AU) and reduce costs.^6^ In this study, we evaluated the impact of a RDV formulary restriction on drug utilization patterns and cost at a safety-net community hospital.

## Methods

### Study design and setting

We conducted a retrospective review of AU and ASP data at a 151-bed hospital in Chicago. Our preintervention period was from May 1 to August 31, 2020 and our postintervention period was from September 1, 2020 to February 28, 2022 (Supplement 1). Our facility has one infectious diseases (ID) physician and five full-time pharmacists, including one ASP pharmacist.

### RDV guidelines

Institutional guidelines were established upon arrival of RDV to our formulary on May 10, 2020 (Supplement 1). Guidelines were shared via the hospital intranet and posters in clinical areas. Initially, RDV had no restrictions, and our guidelines recommended broad use based on the Emergency Use Authorization (Supplement 2). In July, our guidelines evolved to reflect changes in the National Institutes of Health (NIH) COVID-19 guidelines and the ACTT-1 study.^1,2^ These updates advised prioritizing RDV use for patients requiring NC against its use for MV (Supplement 2). The guidelines advised against using RDV in MV patients and highlighted uncertainty in the literature regarding its role in HFNC and NIPPV.

#### Intervention

**Figure 1.**
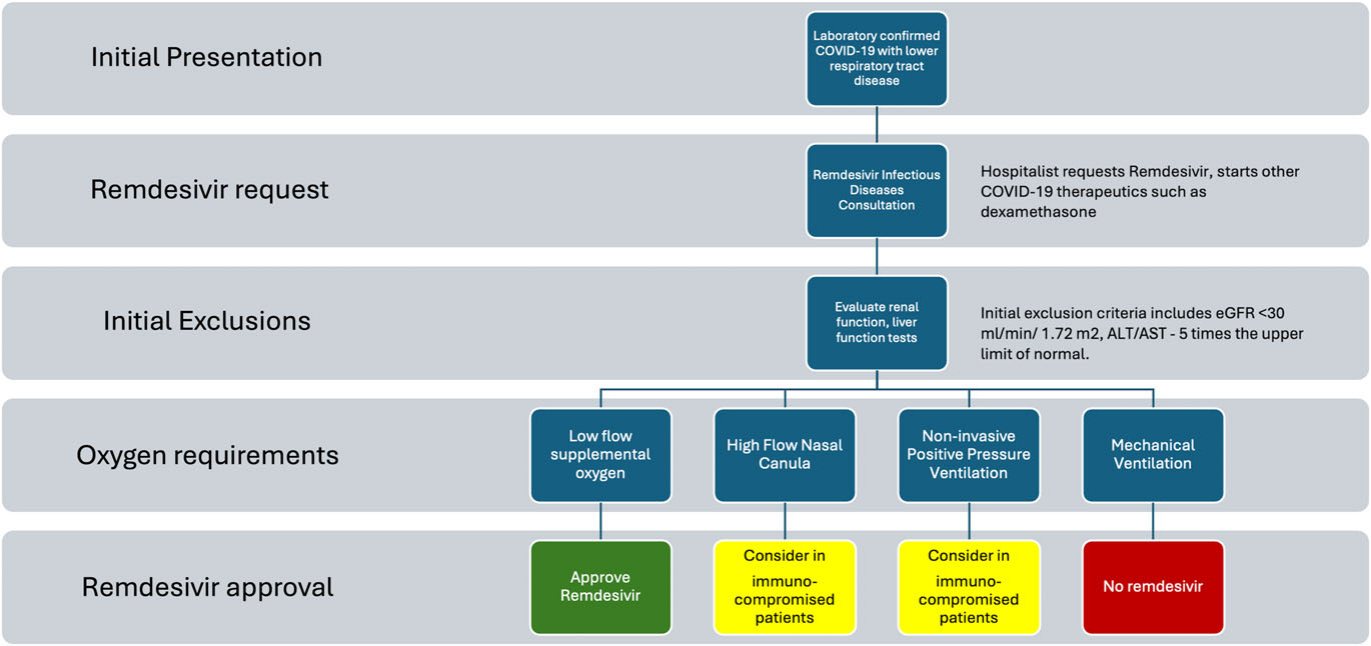
RDV formulary restrictions and approval process.

ASP deployed strict RDV formulary restrictions on September 10, 2020 (Figure 1). To operationalize this restriction, the standard computerized physician order entry for RDV was modified to a “Remdesivir ID evaluation” order to request RDV. This notified the ID physician to evaluate patients for whom RDV was being considered by the primary service. The ID physician then conducted a full consultation and comprehensive patient evaluation to ascertain the appropriateness of RDV therapy, adhering strictly to the NIH guidelines (Supplement 2). RDV dispensation was contingent on ID physician evaluation and approval.

### Data collection and outcome measures

Data on AU and ASP activities from May 1, 2020, to February 28, 2022, were retrospectively reviewed. AU was measured in days of therapy per 1000 patient days (DOT/1000) over time. The preintervention period extended from May 1 to August 31, 2020, while the intervention period spanned from September 1, 2020, to February 28, 2022. ASP prospective audit and feedback (PAF) data was reviewed to assess RDV requests and administrations. RDV avoidance was determined by obtaining the difference between RDV requests and actual RDV utilization. To estimate the predicted DOT/1000 without formulary restrictions, the average monthly DOT/1000 for the intervention period was adjusted proportionally based on the total number of RDV requests. This was calculated by dividing the average monthly DOT/1000 by the number of patients approved for RDV, then multiplying by all RDV requests (Supplement 3). Cost savings were estimated by multiplying the number of instances where RDV was requested and not approved due to guideline discordance by the cost of a five-day RDV course ($3,155).^7^

### Ethics approval

The University of Illinois Chicago Institutional Review Board approved this study.

## Results

### Guideline discordance and cost savings

During the preintervention period, RDV use was guideline-concordant in 31% (19/60) of cases. Had formulary restrictions been in place during this period, missed potential cost savings would have amounted to $129,355 across 41 cases. During the intervention period, 442 RDV evaluation requests occurred, of which 236 (51%) were found to be guideline-concordant following ID consultation. Formulary restrictions in 206 cases (49% not guideline-concordant) led to estimated cost savings of $649,930 across 206 cases.

### RDV utilization patterns

**Figure 2.**
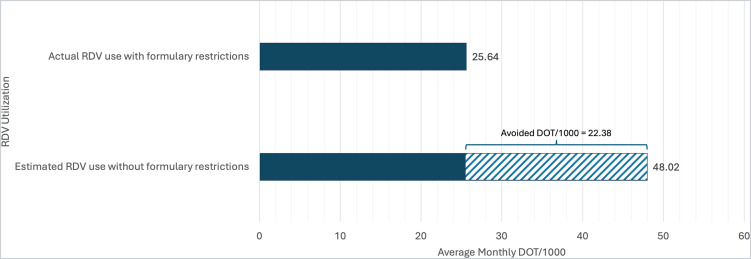
Average monthly DOT/1000 for RDV during our intervention and estimated DOT/1000 without formulary restrictions.

The average monthly preintervention DOT/1000 for RDV was 24.77. DOT/1000 increased during COVID-19 surges, peaking at 54.76 in May 2020 (Supplement 4). Postintervention, the average monthly DOT/1000 was 25.64, peaked at 54.7 during the second wave (October 2020–February 2021), 42.3 during the third wave (March–June 2021), 22.61 during the delta wave (July 2021-November 21) and 48.29 during omicron (December 2021–February 22). Based on the average monthly DOT/1000 for RDV during the intervention period (25.64 for 236 RDV requests), an estimated DOT/1000 of approximately 48.02 could be expected for 442 RDV requests had formulary restrictions not been in place. This corresponds to a total avoided DOT/1000 of 22.38, an 87.29% difference between actual and expected use (Figure 2). The gap between actual and expected monthly DOT/1000 narrowed with each wave except Omicron (Supplement 5–6).

## Discussion

Formulary restrictions led to a reduction in RDV use, increased guideline adherence, and reduced costs. Our interventions led to an estimated reduction in average monthly DOT/1000 of 22.38 and substantial cost savings. The intervention likely had an educational impact and changed prescriber patterns over time, improving compliance each wave except Omicron. (Supplement 5-6). A similar intervention at a 1,041-bed hospital resulted in cost savings of $2.2 million.^8^ This demonstrates the feasibility of this intervention in both small and large hospitals, highlighting the critical role of ASP and formulary restrictions for pandemic response.

Deploying new therapies during pandemics can be challenging. Evolving literature and conflicting guidelines can lead to controversies in treatment strategies. A nationwide survey found a wide gap between evidence-based guidelines and reported RDV use.^9^ This gap was wider in hospitals without formulary restrictions. In contrast, hospitals with restrictions prioritized RDV for patients requiring NC, a trend that increased with each subsequent COVID-19 wave. Restrictions for costly therapies, such as IL-6 inhibitors and JAK-2 inhibitors, were also common during the pandemic.^10^ Our experience mirrors these findings, underscoring the importance of formulary restrictions in guiding COVID-19 treatment strategies and its ability to adapt to evolving data.

Demand outstripped RDV supply early in the pandemic, making allocation strategies and evidence-based use imperative. Unrestricted access to RDV could lead to use in scenarios lacking evidence of benefit, imposing steep expenses on both patients and healthcare systems. At a cost of $3,120 per course, the financial burden on healthcare facilities during waves can become substantial. COVID-19 surges often coincided with reduced procedure reimbursements, higher costs for personal protective gear, and rising staffing costs. Safety-net hospitals are often financially vulnerable, underscoring the importance of formulary restrictions for expensive medications during the pandemic.

Our study has several limitations, including its single-center retrospective design. Our intervention may have inadvertently led to clinicians over-relying on ID review, potentially diminishing their independent evaluation of RDV appropriateness. The absence of interrupted time series analysis, which could have accounted for secular trends, is another limitation. However, we report the feasibility and effectiveness of formulary restrictions in optimizing novel therapeutics during pandemics in smaller hospitals. Over 70% of US hospitals have under 200 beds.^6^ Thus, finding effective stewardship strategies in small and critical access hospitals is important. Lessons learned from this study may have implications for future pandemic preparedness and response.

## Supporting information

Mena Lora et al. supplementary material Mena Lora et al. supplementary material

## Supplementary material

For supplementary material accompanying this paper visit http://doi.org/10.1017/ash.2024.438. click here to view supplementary material
