# Supplementary material for: Impact of a remdesivir formulary restriction by antimicrobial stewardship on drug utilization and cost at a safety-net community hospital during the COVID-19 pandemic
Source: Antimicrob Steward Healthc Epidemiol. 2024 Oct 7;4(1):e164. doi: 10.1017/ash.2024.438 (PMC11474786; doi:10.1017/ash.2024.438)

**SUPPLEMENTS**

**Supplement 1**. Timeline of key events and study interventions during the COVID-19 pandemic response


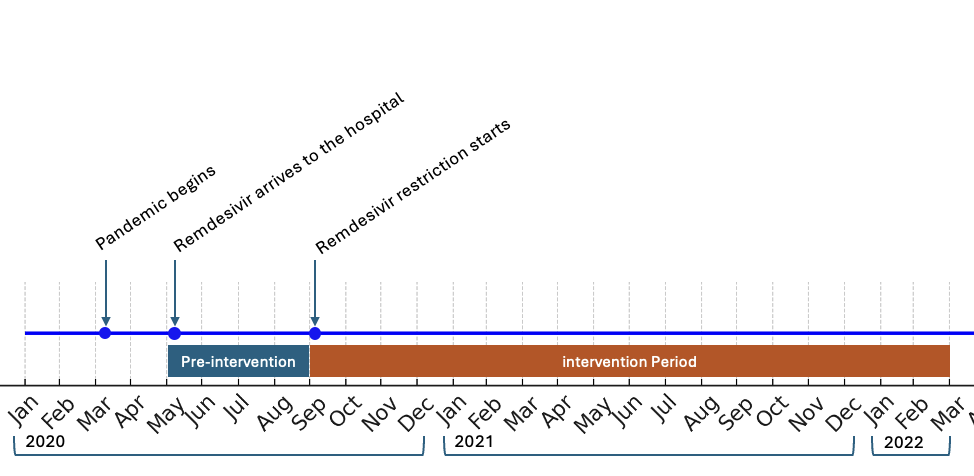


**Supplement 2**. Institutional guidelines at our hospital throughout the pandemic

| **Date** | **Guideline** |
| --- | --- |
| May 14 | **FDA**  Exclusion criteria: eGFR ≤ 30 ml/min/1.72 m2, ALT/AST > 5 x the upper limit of normal, full term neonates (= day to = 28 days old) with a serum creatinine greater than or equal to 1 mg/dL unless the potential benefits outweighs the potential risks.  Inclusion criteria: Per U.S. FDA emergency use authorization for hospitalized children and adults with confirmed or suspected severe COVID-19 Sa02 less than or equal to 94 percent on room air, requiring supplemental oxygen, patients expected to survive the next 48 hours.  Remdesivir is recommended for those with severe COVID-19 on supplemental oxygen rather than in patients on high-flow oxygen, noninvasive ventilation, mechanical ventilation, or ECMO and with shorter onset of symptoms.  **Institutional guideline**  Remdesivir is optimal within the first 10 days of symptom onset |
| July 24 | **Institutional guideline**  Remdesivir is optimal within the first 10 days of symptom onset  Remdesivir is recommended for those with severe COVID-19 on supplemental oxygen rather than in patients on high-flow oxygen, noninvasive ventilation, mechanical ventilation, or ECMO and with shorter onset of symptoms.  **NIH Guidelines**  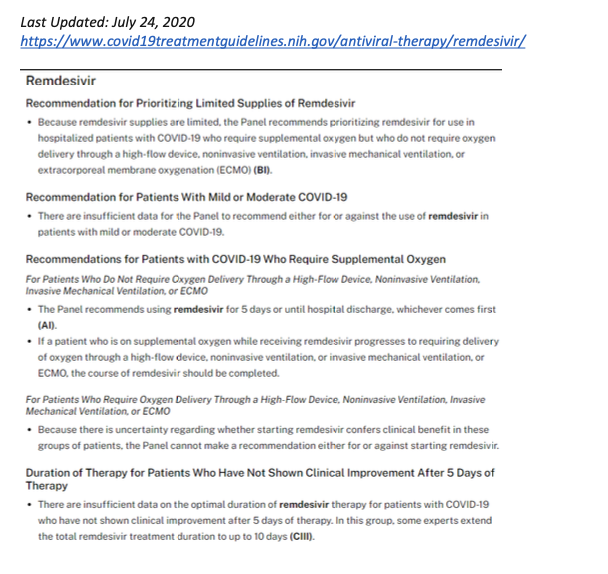 |
| September 1 | **Institutional Guidelines**  Remdesivir is recommended for those with severe COVID-19 on supplemental oxygen rather than in patients on high-flow oxygen, noninvasive ventilation, mechanical ventilation, or ECMO and with shorter onset of symptoms.  Remdesivir formulary restrictions now implemented.  **NIH Guideline**  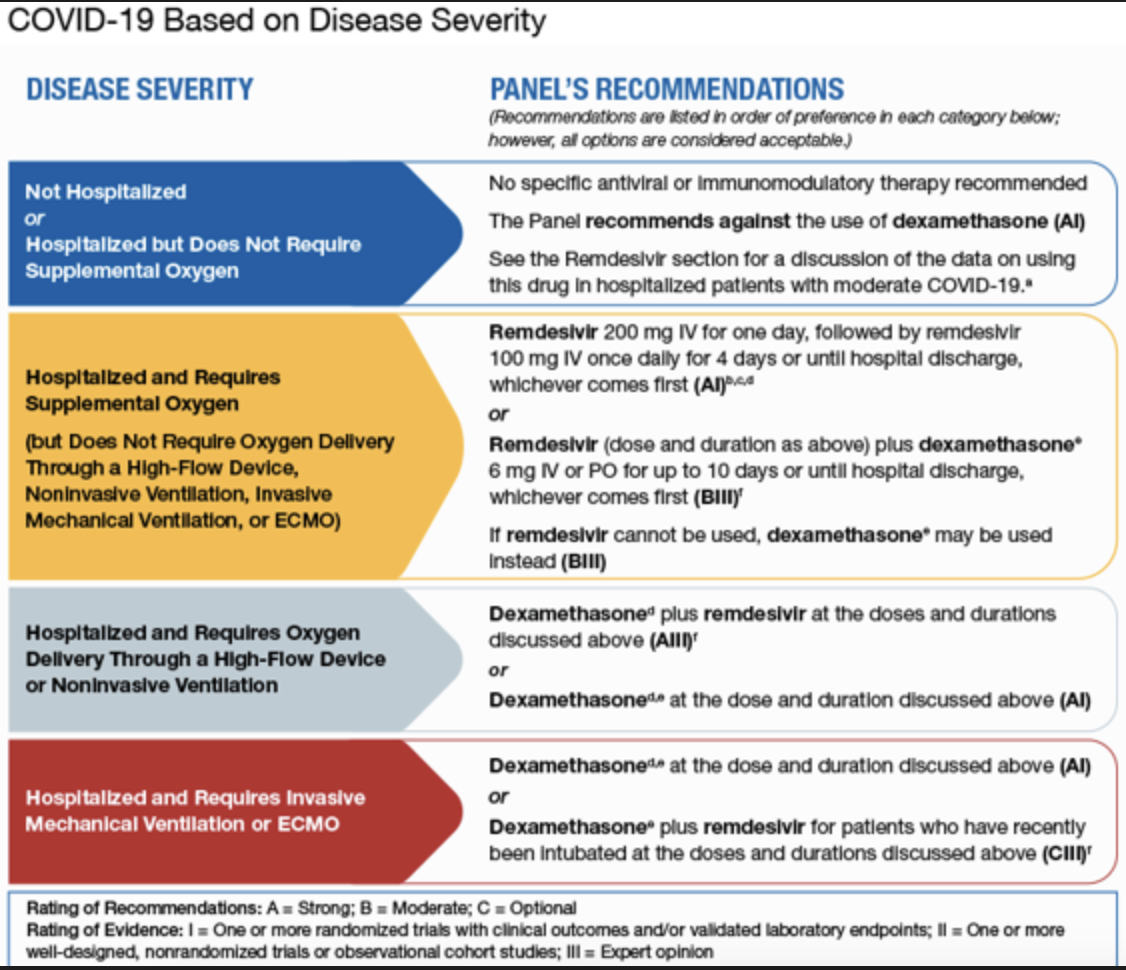 |

**Supplement 3.** Formula for estimated DOT/1000 for RDV without formulary restrictions

Estimated DOT/1000 = ( $\frac{Average Monthly DOT/1000 for RDV}{Patients approved to receive RDV}) x Total RDV Requests$

48.02= ( $\frac{25.64}{236}) x 442$

**Supplement 4.** Average monthly and peak DOT/1000 for RDV during each COVID-19 wave


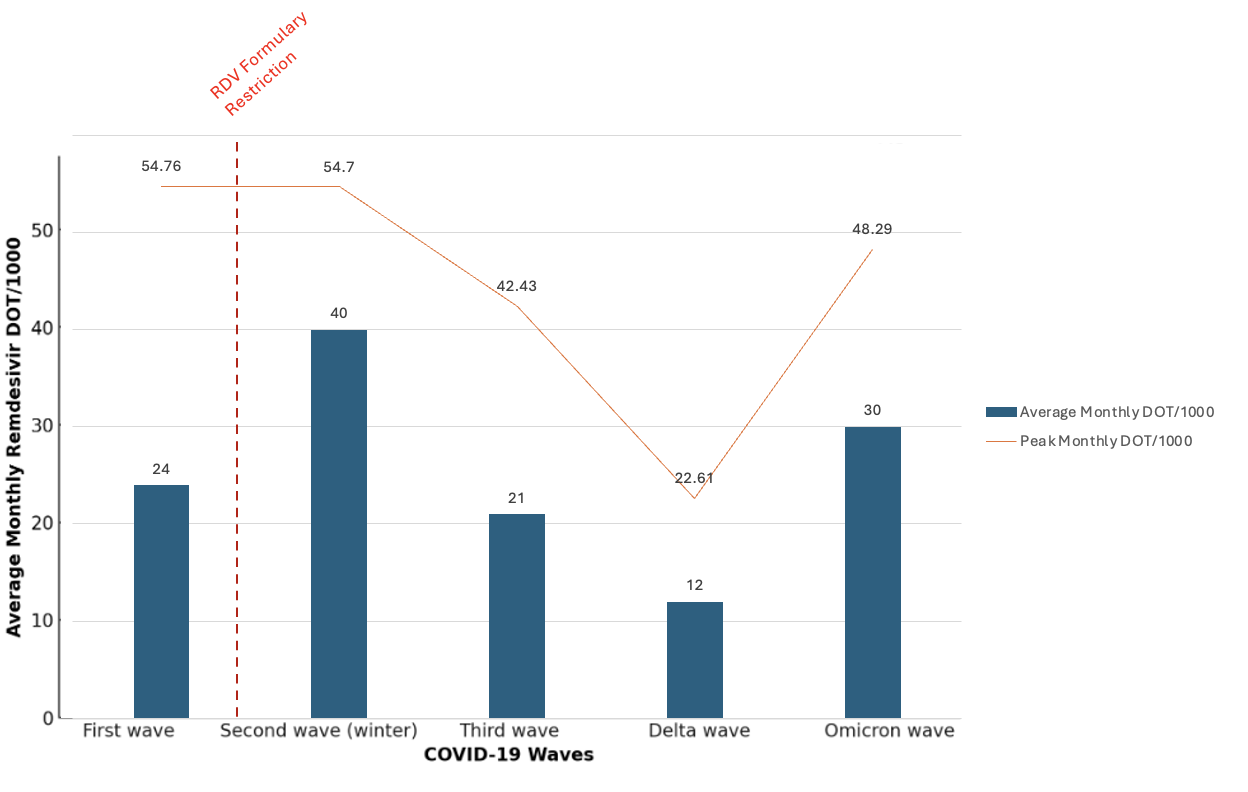


**Supplement 5.** Average monthly DOT/1000 with restrictions and expected monthly DOT/1000 without restrictions each COVID-19 wave.


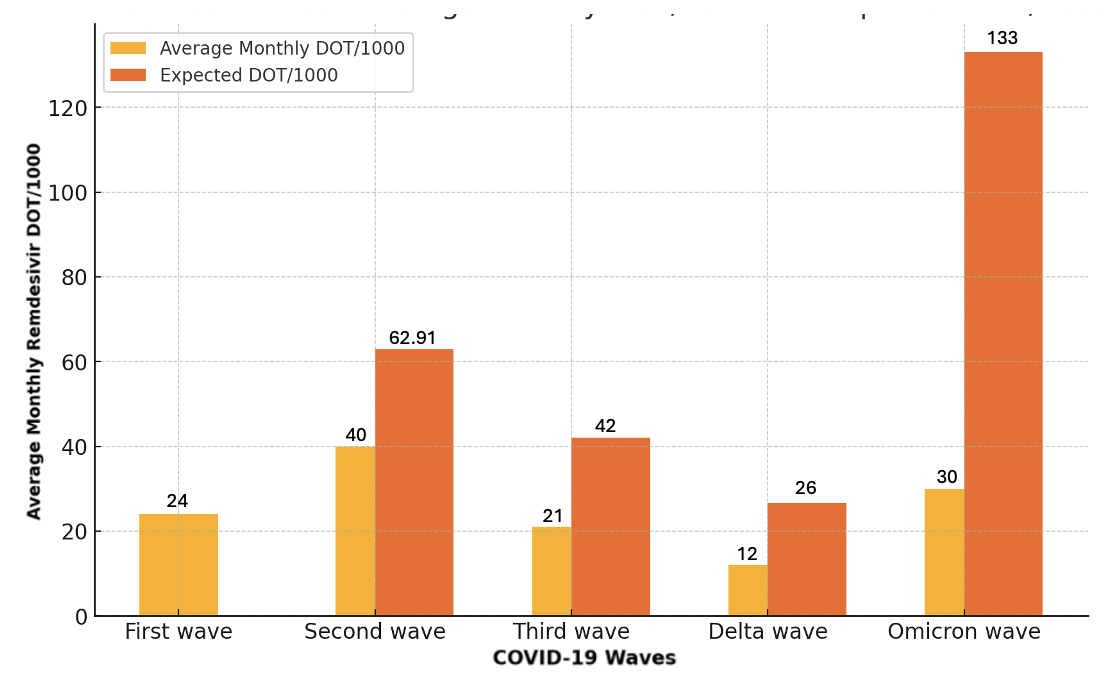


**Supplement 6.** Delta between average monthly DOT/1000 with restrictions and expected monthly DOT/1000 without restrictions each COVID-19 wave.


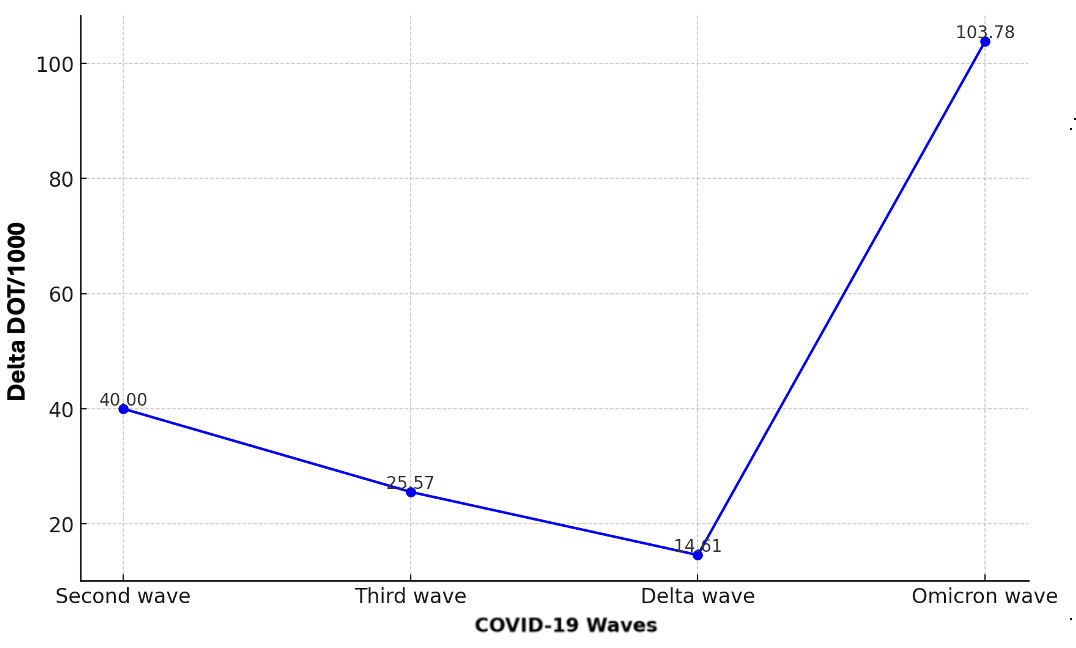

Supplement: Mena Lora et al. supplementary material [file S2732494X24004388sup001.docx]
